# Death from Delivering a Woman with Typhus Fever

**Published:** 1853-07

**Authors:** 


*Death from Delivering a Woman with Typhus fever.*—In the course of his duty as physician-accoucheur to the Royal Pimlico Dispensary, Dr. Fredericke Robert Manson was called upon to deliver a poor woman suffering from typhus fever. Dr. Manson had at the time a slight wound in the hand; through this wound the poisonous element was absorbed, erysipelas supervened, and in nine days after this occurrence his death took place. We have been informed that the patient who was the innocent cause of Dr. Manson's distressing end has herself succumbed; her nurse has shared the like fate, and it is apprehended that the list of victims is not yet complete. —*Ibid.*

